# Increase of light aromatics space time yield by addition of SAPO-34 into multifunctional catalyst

**DOI:** 10.1039/d5ra08666c

**Published:** 2026-01-05

**Authors:** Shiyu Liu, Qiuyun Huang, Jie Wang, Weihua Shen, Yunjin Fang

**Affiliations:** a State Key Laboratory of Chemical Engineering and Low-Carbon Technology, School of Chemical Engineering, East China University of Science and Technology Shanghai 200237 China whshen@ecust.edu.cn yjfang@ecust.edu.cn +86-21-64252829

## Abstract

In this research, SAPO-34 were added into 6Mn4Zr/H-ZSM-5 catalyst and the tri-components catalyst was then granule mixed with ZnCrO_*X*_, which is regarded as methanol synthesis component. The introduction of SAPO-34 could facilitate the accumulation of HCPs and promote conversion of CO. As methanol & DME were preferentially consumed over HCPs in SAPO-34, the selectivity of light aromatics benzene, toluene and xylene (BTX) increased by inhibiting further methylation. The addition of Zn_*X*_Cr could further remarkably enhance the conversion of CO because of it high activity in methanol & DME synthesis, and thus increase the space time yield of BTX components. The influence of Zn_*X*_Cr composition, amount of SAPO-34 and 6Mn4Zr on catalyst performance were investigated. The reaction conditions were further optimized to achieve both high CO conversion (41.51%) and BTX selectivity (31.37%). The space-time yield of BTX could achieve 105.90 mg g_cat_^−1^ h^−1^. The stability of optimized catalyst under optimal reaction condition were evaluated, after 100 h reaction, CO conversion could still reach 36.49% with BTX space time yield of 76.36 mg g_cat_^−1^ h^−1^.

## Abstract 2

SAPO-34 were added into 6Mn4Zr/H-ZSM-5 catalyst and the tri-components catalyst was then granule mixed with methanol synthesis component ZnCrO*__X__*. Addition of SAPO-34 increased selectivity of BTX, and addition of ZnCrO__*X*__ promoted CO conversion.

## Graphical abstract

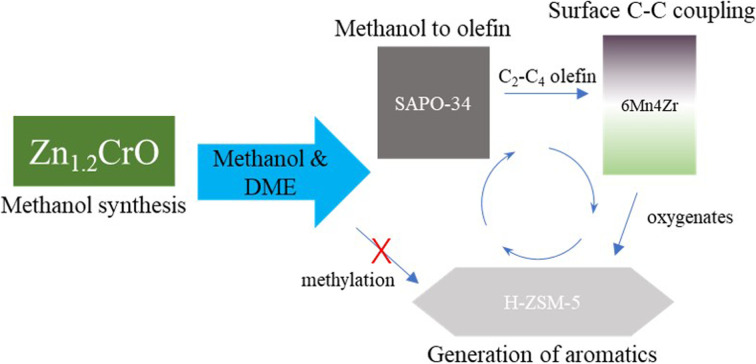

## Introduction

The conversion of syngas to value added chemicals has drawn both academic and industrial interests. A novel route named as oxide-zeolite (OX-ZEO) was proposed by Bao *et al.*,^1,2^ and achieved great success in selective conversion of syngas to olefin (C_2_–C_4_ olefins, named as STO)^1,2^ or aromatics (STA).^3–5^ Generally, the reaction mechanism of OX-ZEO strategy could be concluded as a tandem pathway. CO was firstly converted into intermediates including methanol and dimethyl ester (DME) over oxides, and then the intermediates subsequently diffuse into zeolite for further conversion. The zeolites applied in OX-ZEO strategy for production of light olefin are usually SAPO-34, AlPO-18, *etc.*; while when generating aromatics (STA), ZSM-5 could be applied.^1–3,6–8^

In STA, methanol and DME serve as the primary intermediates generated over oxide components, leading to relatively low selectivity toward light aromatics (*e.g.*, benzene, toluene, and xylene, summarized as BTX) in the conventional OX-ZEO strategy.^9–12^ Such limitation arises from the methylation of light aromatics at the external acid sites of H-ZSM-5, which produces heavier products (*e.g.*, trimethylbenzene and tetra-methylbenzene). To achieve higher selectivity of light aromatics, silanization of zeolite was applied to eliminate the external acid sites.^13,14^ Moreover, H-ZSM-5 with shorter length in *b* axis could promote diffusion of light aromatics thus also prevent the methylation mentioned above.^15^ Except for modification of H-ZSM-5, introducing SAPO-34 into OX-ZEO strategy could also avoid the generation of higher aromatics, which would be benefitted from the preferential conversion of intermediates in SAPO-34 rather than H-ZSM-5.^16^

Early studies on STA were constrained by low CO conversion.^1,4,7,13,17^ To improve conversion, the activation ability of H_2_ and CO should be strengthened. Addition of H_2_ activation species (*e.g.*, ZnO or MoO_*X*_) could directly enhance the activation of H_2_, and further improve the generation of oxygen vacancy (O_V_),^1,7^ however, the selectivity of aromatics would decrease even at little addition amount. Besides, constructing single-atom dispersed Fe into ZnCr spinel could increase the CO conversion without decrease in aromatics selectivity.^18,19^ For single-atom dispersed Mo-ZnCrO_*X*_, the coordination condition of Mo atom also exhibited significant influence on catalytic activity.^20^ The decrease of aromatics selectivity could be associated to over-hydrogenation, which was further confirmed by the loss of hydrocarbon pool species (HCPs) inside H-ZSM-5.^21^ A comparison of STA and STO reveals that oxide components in STO contain higher proportions of H_2_-activating species, resulting in substantially higher CO conversion. This difference suggested that SAPO-34 (used in STO) could inhibit loss of HCPs, in contrast to H-ZSM-5 (used in STA).

In this work, ZnCrO_*X*_ with different Zn/Cr ratio were prepared as methanol synthesis components. When combined with STA catalyst (6Mn4Zr/H-ZSM-5), CO conversion increased with higher Zn/Cr ratio with decreasing in aromatics selectivity; while when combined with STO catalyst (6Mn4Zr/SAPO-34), a limitation in CO conversion were observed and selectivity of light olefin maintained similar value. Further, 6Mn4Zr, H-ZSM-5 and SAPO-34 were coupled simultaneously to enhance selectivity of BTX. When mixed with ZnCrO_*X*_, the ternary catalyst with higher proportion of SAPO-34 could achieve higher CO conversion. The total aromatics selectivity decreased with increasing Zn/Cr ratio, while increasing proportion of SAPO-34 could alleviate such decrease. Thermogravimetry analysis convinced that addition of SAPO-34 could maintain HCPs, and gas chromatograph-mass spectrometer revealed the evolution of HCPs composition with the addition of SAPO-34.

## Experimental

### Chemical and physical properties of catalysts

#### Materials

The deionized water was homemade, and other chemicals (AR, 99.7% in purity) were purchased from Shanghai Titan Scientific Co., Ltd.

#### Synthesis of catalysts

6Mn4Zr and ZnCr spinel oxides were synthesized by co-precipitation method. In general, a mixed solution of metal nitrates (1 M in total) was co-fed with (NH_4_)_2_CO_3_ (1 M). The pH value at the end of co-precipitation was maintained at 7. After aging at 70 °C for 3 h, the precipitates were filtrated and washed for 6 times by deionized water. The solids were dried at 100 °C overnight, and then calcinated at 550 °C for 6 h. The binary oxides were denoted as *x*Mn*y*Zr, where *x*, *y* represent their molar ratio. For ZnCr spinel, it was denoted as Zn_*a*_Cr, where *a* represents the mole ratio of Zn/Cr.

H-ZSM-5 with short *b*-axis were synthesized by hydrothermal method with urea addition. The Si/Al ratio of H-ZSM-5 was fixed at 60. The synthesis method was referred to our previous work.^22^ SAPO-34 with Si/Al ratio of 0.1 were prepared by hydrothermal treatment method. The composition of synthesis gel was referred to work of Sun.^23^

#### Mixing method of catalysts

Powder mixing method and granule mixing method were applied in this research. For powder mixing method, certain amounts of components were added were first grinded in an agate mortar till uniform, then tableted and crushed into 40–80 meshes. “/” were applied as separator between components in the same particle. The mass ratio was listed in brackets following composition of particle.

For granule mixing method, each particle (40–80 meshes) was prepared separately. The particles with certain amount were loaded into quartz tube then shake into uniform, “|” were applied as separator between particles. If one particle composed of more than one component, the components should be enclosed in brackets. Mass ratio of each particle were listed in brackets just following the particles donation with italic.

For catalyst denoted as Zn_1.2_Cr(1)|(6Mn4Zr/H-ZSM-5/SAPO-34)(2 : 1 : 1)(8), it represents the first particle composed of Zn_1.2_Cr, and the second particle composed of 6Mn4Zr, H-ZSM-5 and SAPO-34. Mass ratio between Zn_1.2_Cr and (6Mn4Zr/H-ZSM-5/SAPO-34) was 1 : 8, and the mass ratio of 6Mn4Zr : H-ZSM-5 : SAPO-34 is 2 : 1 : 1.

#### Catalytic evaluation

Catalyst evaluation was performed in a stainless steel fixed-bed reactor with quartz tube lining. Typically, 1.2 g catalyst was loaded in the quartz tube. Before reaction, the catalyst was reduced under H_2_ at 400 °C for 2 h. After the reactor was cooled down to room temperature, the atmosphere was switched to syngas and the pressure was raised to desired value; the reactor was then heated. The space velocity of syngas (4% N_2_ as internal standard and H_2_/CO ratio of 2) was kept at 3000 mL g_cat_^−1^ h^−1^ for STA reaction generally, while it was kept at 6000 mL g_cat_^−1^ h^−1^ for methanol synthesis. The products were analyzed by online gas chromatograph (GC2060, Shanghai Ruimin, China). One TCD detector connected with TDX-01 column was used to quantify N_2_, CO, CH_4_ and CO_2_; one FID detector (FID1) with Restek Rt-Q-Bond column was used to quantify light hydrocarbons, methanol and dimethyl ether (DME); another FID detector (FID2) with KB-PONA column was used to quantify heavy hydrocarbons. The CO conversion and CO_2_ selectivity was calculated from TCD results. CO conversion was calculated as follow.
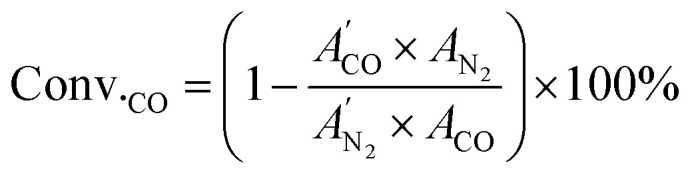
where 
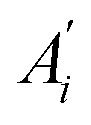
 represents area of *i* component detected in TCD detector before reaction, while *A*_*i*_ represents area of *i* component detected in TCD detector during reaction.

Selectivity of CO_2_ was calculated as follow.
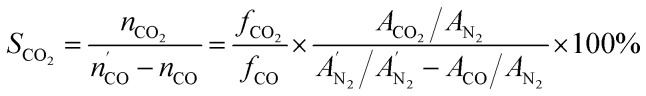
where *f*_*i*_ represents the molar corresponding factor of *i* component to N_2_, and was detected before reaction.

The organic products selectivity was calculated by normalization of the two FID detector (without considering CO_2_):
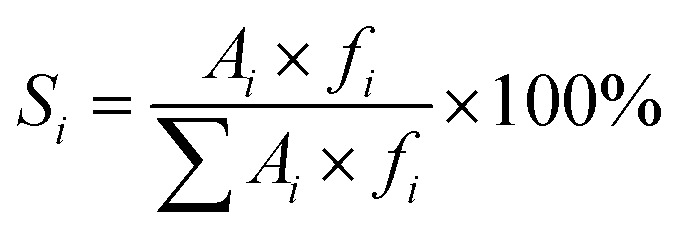


Carbon balance was calculated as the ratio of the outlet carbon atoms to the inlet carbon atoms. CH_4_ was chosen as link molecular between TCD and FIDs to calculate the carbon balance, the methane amount was calculated in the same equation as CO_2_. All the carbon balance in this work were between 95 and 105%.

The blank reaction was performed and the CO conversion was less than 0.1%, with almost only methane in products. Thus, the reaction results over catalysts were not excluded with this minor difference.

The reaction results were obtained at time on stream (TOS) of 10 h.

#### Characterizations

X-ray powder diffraction (XRD) patterns were obtained using a Rigaku D/max 2550 X-ray Diffractometer (Rigaku, Japan) with Cu Kα radiation (*λ* = 0.154 nm). The instrument was operated at 40 kV and 100 mA. The scanning speed was generally set at 10° min^−1^.

X-ray fluorescence (XRF) was performed using a PANalytical Zetium spectrometer (PANalytical, Netherlands).

X-ray photoelectron spectroscopy (XPS) was performed using a Thermo Scientific K-Alpha instrument (Thermo Scientific, America). The binding energy was corrected using C 1s = 284.80 eV. Each sample was pre-reduced under H_2_ atmosphere for 1 h prior to the XPS measurement.

H_2_ temperature programmed reduction (H_2_-TPR) and NH_3_ temperature programmed desorption (NH_3_-TPD) were performed on a VDSORB-91i standard chem-sorption station (VODO, China). For H_2_-TPR, about 30 mg of sample was loaded in U type tube. And the sample was pre-treated under Ar for 2 h at 200 °C. For NH_3_-TPD, about 50 mg of sample was loaded in U-type tube. Before adsorption, the sample was pre-treated under He for 2 h at 200 °C. After desorption, several pulses of NH_3_ were injected into the sample for quantitative analysis.

Thermogravimetric analysis (TG) was conducted using the NETZSCH STA 449 F3 Jupiter (NETZSCH, Germany). Around 50 mg of sample were applied in TG experiment. The atmosphere of TG experiment was set as air and the heating rate was set to 10 °C min^−1^.

The morphology of materials was obtained using a FEI Nova NanoSEM 450 Vacuum Scanning Electron Microscopy (SEM) at an acceleration voltage of 15 kV. All the samples were coated by Pt for 90 s before characterization.

## Results and discussion

### Chemical and physical properties of catalysts

**Fig. 1:**
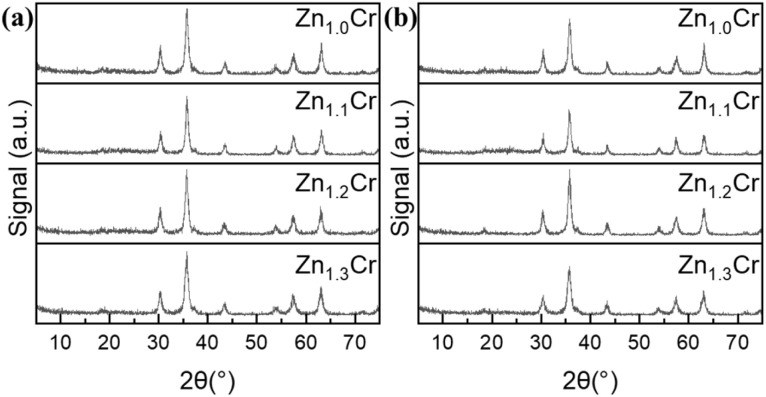
XRD patterns of Zn_*X*_Cr (a) fresh oxides; (b) spent oxides.

The composition of oxides and zeolites were obtained by XRF and listed in Table S1. The detected composition of materials is generally in accordance with the label value. XRD patterns related to 6Mn4Zr, H-ZSM-5 and SAPO-34 were shown in Fig. S1(a). As demonstrated in our previous research, both Mn_2_O_3_ and Mn_0.2_Zr_0.8_O_1.8_ were found in 6Mn4Zr. The H-ZSM-5 and SAPO-34 shows typical MFI and CHA diffraction peaks, respectively. During reaction, the Mn_2_O_3_ was reduced to MnO, which could be convinced by diffraction pattern of 6Mn4Zr/H-ZSM-5 after reaction. As urea were added in the synthesis recipe of H-ZSM-5, the morphology of H-ZSM-5 was found be sheet-like, as being shown in Fig. S1(b); for SAPO-34, the typical cubic shape could be observed in Fig. S1(c). The XRD patterns related to Zn_*X*_Cr were shown in Fig. 1. For the fresh Zn_*X*_Cr oxides, only phase of ZnCr_2_O_4_ were found. The absence of ZnO peaks could be ascribed as good solubility of ZnO in ZnCrO_4_ and the diffraction peaks were nearly not changed after reaction.

**Fig. 2:**
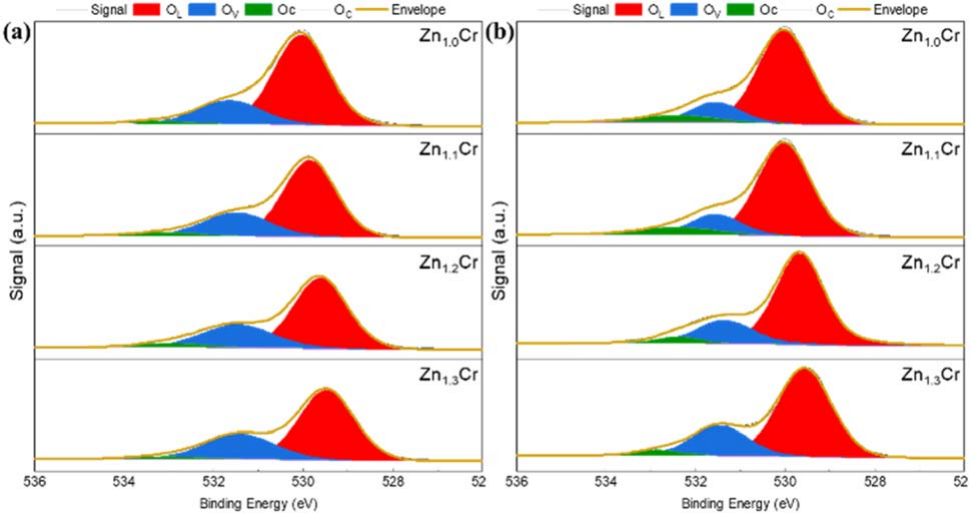
XPS profile of Zn_*X*_Cr (a) fresh oxides; (b) spent oxides.

The reducibility of Zn_*X*_Cr was investigated by H_2_-TPR and shown in Fig. S2. Several peaks could be found in the TPR profiles, centering at 250 °C, 350 °C and 400 °C. The peak centered at 250 °C dominate the reduction process, and both its area and strength increased with addition of Zn. Neither ZnCr_2_O_4_ nor additional dissolved ZnO could be reducible, thus the peaks should be related to generation of surface oxygen vacancies. To verify it, Zn_*X*_Cr samples before and after reaction were collected and performed XPS experiment. As shown in Fig. 2, The O 1s orbit could be separated into 3 parts, which is lattice oxygen (O_L_) at the binding energy of 528.5–530.5 eV, the peak of vacancy oxygen (O_V_) at 530.5–531.5 eV and the peak of chemisorbed oxygen (O_C_) at 531.5–533 eV.^23–27^ The O_V_ over surface of reduced Zn_*X*_Cr in Fig. 2b is much higher than fresh Zn_*X*_Cr in Fig. 2a. The quantitative results were shown in Table S2, it was found that the Ov concentration over oxides surface were all remarkably increased after reduction. Moreover, the surface Zn/Cr ratio was also increased, indicating that the reduction atmosphere could segregate Zn^2+^ from bulk to surface and thus enhance the activation of H_2_ which is benefitted the generation of O_V_.

The acid property of H-ZSM-5 and SAPO-34 were studied by NH_3_-TPD (shown in Fig. S3) and the quantification analysis were listed in Table S3. The acid sites could be separated into three parts for each zeolite. The peak centered around 100 °C is ascribed as desorption peak of weak acid site; the peak centered at around 160 °C is desorption peak of medium strong acid site; and the peak centered at above 400 °C is desorption peak of strong acid site.^28^ Quantification results of NH_3_-TPD are listed in Table S3, the total acid density of SAPO-34(720.41 µmol g^−1^) was much higher than that of H-ZSM-5(73.45 µmol g^−1^); while the proportion of weak acid sites of SAPO-34 (43.01%) was higher than H-ZSM-5(19.07%).

### Increasing BTX proportion by addition of SAPO-34

Catalyst composed of 6Mn4Zr, H-ZSM-5 and SAPO-34 were first prepared with weight proportion of 6Mn4Zr fixed at 50%. As shown in Table 1, when 6Mn4Zr were coupled with H-ZSM-5, CO conversion achieved 15.11% with total aromatics selectivity of 84.77%. However, even sheet-like H-ZSM-5 were applied, the BTX selectivity reached only 12.73%, which is a relative low value. While for 6Mn4Zr/SAPO-34, the main products were light olefins (82.43%), and CO conversion was only 12.76%. CO conversion slightly increased when SAPO-34 were coupled with 6Mn4Zr along with H-ZSM-5, and it reached maximum value of 16.13% when the mass ratio of H-ZSM-5 to SAPO-34 was 2 : 1, however, the BTX selectivity maintained similar value with 6Mn4Zr/H-ZSM-5 at this ratio and aromatics selectivity decreased to 71.08%. Further increasing the ratio of H-ZSM-5 : SAPO-34 to 1 : 1, CO conversion decreased to 45.60%, the total selectivity of aromatics decreased to 66.65% with BTX selectivity increased to 34.63%. Moreover, the BTX proportion in total aromatics reached 52.75%. For 6Mn4Zr/H-ZSM-5/SAPO-34(3 : 1 : 2), CO conversion, total aromatics selectivity, BTX selectivity and proportion of BTX in aromatics all decreased.

**Table 1:** Reaction results over 6Mn4Zr/H-ZSM-5/SAPO-34 with different mass ratio

Mass ratio of H-ZSM-5 to SAPO-34^a^	CO conversion (%)	CO_2_ selectivity (%)	Organic products selectivity (%)						BTX ratio in aromatics (%)
			CH_4_	Methanol & DME	C_2_–C_4_ paraffin	C_2_–C_4_ olefin	C_5_^+^	Aromatics (BTX)	
1 : 0	15.11	43.86	2.74	—	3.56	3.26	5.67	84.77 (12.73)	15.07
2 : 1	16.13	39.65	5.09	0.06	16.16	4.43	6.13	68.12 (12.81)	18.80
1 : 1	15.60	40.13	4.73	0.06	15.82	6.07	6.65	66.65 (34.63)	52.75
1 : 2	14.97	39.48	5.62	0.07	15.02	8.48	4.45	66.36 (33.04)	49.78
0 : 1	12.76	39.57	7.85	0.34	6.13	82.43	3.25	—	

a 6Mn4Zr were kept as 50 wt% in catalyst. Reaction condition: 400 °C, 3 MPa, H_2_/CO = 2, 3000 mL g_cat_^−1^ h^−1^.

**Fig. 3:**
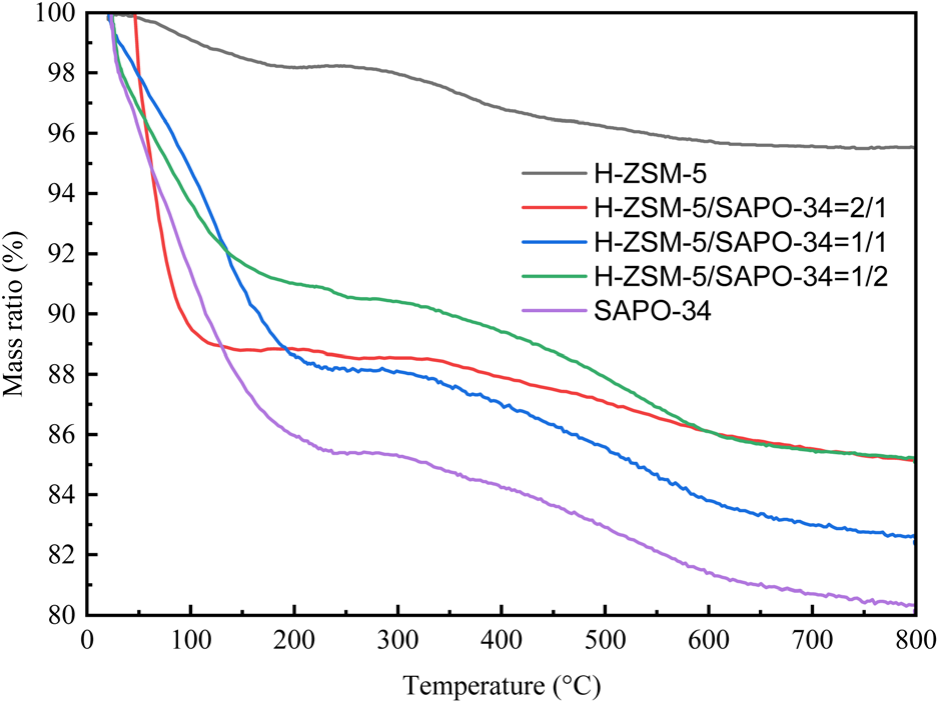
TGA profile of spent catalyst.

In our previous research,^21^ the amounts of accumulated HCPs could influence CO conversion by promoting consumption of intermediates. Besides, SAPO-34 exhibited much higher accumulation amount of HCPs compared with H-ZSM-5. Herein, to reveal the impact of addition of SAPO-34 on STA reaction, TG analysis were performed for the spent triple-components catalysts, and the profiles were shown in Fig. 3. The mass loss in TG profile were separated into two parts, the mass loss before 250 °C was regarded as escape of water, while the following mass loss should be ascribed as consumption of HCPs inside zeolites. Moreover, a slight increase in mass ratio at around 230 °C could be found in profiles, which should be ascribed as re-oxidization of Mn^2+^ into Mn^3+^. As the ratio of 6Mn4Zr in all catalyst was maintained the same value, thus the influence of such re-oxidization could be neglected. Amounts of HCPs were calculated as difference between residual mass at 250 °C and 800 °C, and detailed data were shown in Table S4. The least HCPs was accumulated on 6Mn4Zr/H-ZSM-5, which is only 2.71%. With the addition of SAPO-34, the amounts of HCPs increased to 3.52% over 6Mn4Zr/H-ZSM-5/SAPO-34(3 : 2 : 1), and reached maximum value of 5.72% over 6Mn4Zr/H-ZSM-5/SAPO-34(2 : 1 : 1). However, the amount of HCPs decreased with further addition of SAPO-34, which indicated that the generation of HCPs was not merely affected by the pore size of zeolite.

The pore size of SAPO-34 (8-membered ring) is much smaller than H-ZSM-5 (10-membered ring), thus more HCPs could accumulate in channel of zeolite of 6Mn4Zr/SAPO-34 than 6Mn4Zr/H-ZSM-5. Moreover, the smaller pore size also increased diffusion resistance of intermediates, thus caused decrease in CO conversion. However, CO conversion could increase at a lower addition of SAPO-34, which is against the influence diffusion. The HCPs were first proposed in studies of methanol to hydrocarbons researches, including both methanol to olefin (MTO) and methanol to aromatics (MTA). An induction period was generally reported in researches of MTH, during when methanol conversion and unsaturated hydrocarbons selectivity increased with prolonging reaction time, and generated HCPs. The HCPs was regarded as co-catalyst in MTH reaction, which react with methanol in the dual-cycle mechanism. Methanol could participant in methylation of olefin (olefin cycle) or aromatics (aromatics cycle). The generated olefins could be converted to aromatics by H transfer reaction generating paraffins and aromatics simultaneously.

The increase of CO conversion at lower addition of SAPO-34 should be benefitted from the increase in HCPs amount, which promoted conversion of intermediates; however, at higher addition amount of SAPO-34, consumption of intermediates was inhibited by the higher diffusion resistance of SAPO-34, which decreased CO conversion. Moreover, the existence of H-ZSM-5 could facilitate the generation of aromatics, which might accelerate the generation of HCPs, thus the maximal amounts of HCPs was observed over 6Mn4Zr/H-ZSM-5/SAPO-34(2 : 1 : 1).

As explained in out previous work, 6Mn4Zr could convert CO to methanol & DME.^29^ After addition of SAPO-34, methanol & DME were preferentially consumed in channel of SAPO-34 with exist abundant HCPs, generating light olefins. The light olefins could participant in C–C coupling over 6Mn4Zr, and form C_2_^+^ oxygenates, which could be converted into aromatics in H-ZSM-5; it could also participant in H transfer reaction in H-ZSM-5, also generating aromatics with light paraffin as by-product. As H transfer reaction could occur, the selectivity of total aromatics decreased. BTX selectivity increased because of the preferentially consumption of methanol & DME in SAPO-34.

### Couple Zn_*X*_Cr with 6Mn4Zr/H-ZSM-5/SAPO-34

In our precious research,^21^ methanol synthesis component ZnMnZr were added to enhance the conversion of CO. Herein, rather than ZnMnZr, the Zn_*X*_Cr with different Zn concentration were applied. The reaction results over Zn_*X*_Cr in syngas conversion were listed in Table 2. As the activation ability of H_2_ was improved with increasing concentration of Zn, CO conversion increased with increasing concentration of Zn. Methanol and DME were major products over all Zn_*X*_Cr oxides, while its selectivity decreased with increasing concentration of Zn, moreover, the selectivity of methane also decreased; on the contrary, selectivity of light olefin increased rather than light paraffins (C_2_–C_4_ paraffins). As more H_2_O could be generated when selectivity of methanol and DME decreased, selectivity of CO_2_ increased with Zn concentration. These changes in products selectivity could be affected by the evolution of surface composition.

**Table 2:** Reaction results of syngas conversion over Zn_*X*_Cr^a^

Composition of Zn_*X*_Cr	CO conversion (%)	CO_2_ selectivity (%)	Organic products selectivity (%)				
			CH_4_	Methanol & DME	C_2_–C_4_ paraffin	C_2_–C_4_ olefin	C_5_^+^
Zn_1.0_Cr	2.23	15.89	30.21	64.36	3.01	2.42	0.00
Zn_1.1_Cr	3.06	16.25	20.51	65.95	7.01	6.53	0.00
Zn_1.2_Cr	3.16	18.62	18.52	58.91	6.76	13.89	1.92
Zn_1.3_Cr	3.49	21.80	16.69	55.93	6.50	18.27	2.61

a Reaction condition: 400 °C, 3 MPa, H_2_/CO = 2, 6000 mL g_cat_^−1^ h^−1^.

The Zn_*X*_Cr were then granule mixed with 6Mn4Zr/H-ZSM-5 as STA catalyst and 6Mn4Zr/SAPO-34 as STO catalyst, respectively. The reaction results were listed in Table 3. When Zn_1.0_Cr were mixed with 6Mn4Zr/H-ZSM-5, CO conversion was increased to 18.53% with aromatics selectivity decreased to 80.47%; with increasing Zn/Cr ratio to Zn_1.3_Cr. CO conversion gradually increased to 30.32%, while total aromatics selectivity decreased to 63.21%. As demonstrated in our previous work,^21^ when more methanol & DME were generated from oxide components, the accumulation of HCPs in H-ZSM-5 would be inhibited by H transfer reaction. However, the BTX proportion in total aromatics increased from 15.07% to 24.98%. This could be caused by the C_2_–C_4_ olefins generated over Zn_*X*_Cr, which promoted methanol & DME consumption inside H-ZSM-5 rather than methylation of BTX over external acid sites of H-ZSM-5.

**Table 3:** Reaction results of syngas conversion over Zn_*X*_Cr(1)|6Mn4Zr/H-ZSM-5/SAPO-34(0.5 : *x* : *y*)(8)

Composition of Zn_*X*_Cr	Mass ratio of ZSM-5 to SAPO-34^a^	CO conversion (%)	CO_2_ selectivity (%)	Organic products selectivity (%)						BTX in aromatics (%)
				CH_4_	Methanol & DME	C_2_–C_4_ paraffin	C_2_–C_4_ olefin	C_5_^+^	Aromatics (BTX)	
Zn_1.0_Cr	1 : 0	18.53	43.37	2.73	0.06	9.91	2.71	4.11	80.47 (16.27)	20.22
	2 : 1	19.07	41.14	3.46	0.03	14	4.78	5.48	72.26 (27.55)	38.13
	1 : 1	18.98	41.43	2.85	0.05	14.79	6.21	6.88	69.21 (32.67)	47.20
	1 : 2	18.88	40.98	2.94	0.03	14.35	8.71	8.00	65.98 (34.81)	52.76
	0 : 1	13.29	40.23	3.96	0.24	6.66	85.6	3.54	—	
Zn_1.1_Cr	1 : 0	23.14	42.74	1.85	0.05	14.61	3.22	8.02	72.25 (14.72)	20.37
	2 : 1	24.00	41.69	2.17	0.04	16.4	4.39	8.74	68.26 (24.17)	35.41
	1 : 1	25.96	41.24	1.96	0.02	16.81	5.18	8.48	67.54 (31.55)	46.71
	1 : 2	24.83	40.8	2.05	0.03	16.74	7.08	9.13	64.96 (33.23)	51.15
	0 : 1	15.44	40.29	2.92	0.45	7.99	84.1	4.54	—	
Zn_1.2_Cr	1 : 0	27.50	42.58	1.67	0.04	17.33	2.99	10.48	67.49 (15.38)	22.79
	2 : 1	30.94	40.92	2.33	0.02	20.56	3.78	11.70	61.61 (25.29)	41.05
	1 : 1	30.77	39.88	1.72	0.03	19.87	5.17	11.51	61.7 (29.3)	47.49
	1 : 2	29.66	41.50	1.89	0.02	20.30	6.98	15.30	55.50 (29.95)	53.97
	0 : 1	19.83	41.04	2.55	0.36	5.12	86.5	5.48	—	
Zn_1.3_Cr	1 : 0	30.32	42.61	1.75	0.07	20.62	2.41	11.94	63.21 (15.79)	24.98
	2 : 1	32.79	41.15	1.71	0.05	21.89	3.73	12	60.63 (23.7)	39.09
	1 : 1	33.87	40.74	1.83	0.02	22.97	4.09	14.92	56.16 (28.92)	51.50
	1 : 2	31.68	41.85	1.87	0.03	20.98	7.34	14.87	54.92 (29.06)	52.95
	0 : 1	21.87	40.97	2.32	0.54	4.65	86.93	5.56	—	—

a 6Mn4Zr were kept as 50 wt% in catalyst. Reaction condition: 400 °C, 3 MPa, H_2_/CO = 2, 3000 mL g_cat_^−1^ h^−1^.

While when Zn_*X*_Cr were mixed with 6Mn4Zr/SAPO-34, the main hydrocarbon products were C_2_–C_4_ olefins. With increasing the Zn/Cr ratio, selectivity of C_2_–C_4_ olefins further increased. However, when the same Zn_*X*_Cr were mixed with 6Mn4Zr/SAPO-34 and 6Mn4Zr/H-ZSM-5 respectively, CO conversion over Zn_*X*_Cr|6Mn4Zr/SAPO-34 was always lower than Zn_*X*_Cr|6Mn4Zr/H-ZSM-5, which revealed the importance of diffusion condition on CO conversion. For reaction results over different Zn_*X*_Cr with the same 6Mn4Zr/H-ZSM-5/SAPO-34, CO conversion increased with Zn/Cr ratio. The increase in CO conversion should be benefited from the improvement in methanol & DME synthesis activity at high Zn/Cr ratio. The total aromatics selectivity first increased after addition of Zn_1.0_Cr then decreased with increasing the ratio of Zn/Cr. Addition of Zn_1.0_Cr could provide more methanol & DME available, and more aromatics could be generated, thus, even underwent further conversion like cracking, the total aromatics could reach a much higher level. However, with increasing the Zn/Cr ratio, the generated methanol & DME could not be completely consumed by SAPO-34, thus facilitated H transfer reaction in H-ZSM-5, resulting in generation of by-products C_2_–C_4_ paraffins. The unconsumed methanol & DME could also participant in methylation of BTX at external acid sites of H-ZSM-5, leading to decease in BTX selectivity and could also cause the loss of HCPs in zeolites. Additionally, when coupled with the same 6Mn4Zr/H-ZSM-5/SAPO-34, enhance in CO conversion is less effective when Zn/Cr ratio is higher; moreover, benefitted from accumulation of HCPs, when the same Zn_*X*_Cr were applied, CO conversion over Zn_*X*_Cr|6Mn4Zr/H-ZSM-5/SAPO-34 was always higher than Zn_*X*_Cr|6Mn4Zr/H-ZSM-5. Both the results revealed the importance of HCPs in promoting CO conversion. The reaction mechanism over Zn_*X*_Cr|6Mn4Zr/H-ZSM-5/SAPO-34 was illustrated in Scheme S2. CO mainly activated and converted into methanol & DME over surface of Zn_*X*_Cr as intermediates. The intermediates then diffuse into SAPO-34 and converted by HCPs into C_2_–C_4_ olefins, avoiding methylation of BTX at external acid sites of H-ZSM-5. The olefins could participant in C–C coupling over surface of 6Mn4Zr, it was converted into oxygenated for generation of aromatics in H-ZSM-5. Besides, the olefins could also participant in H transfer reaction in H-ZSM-5 generating aromatics and paraffins as by-products. At higher Zn/Cr ratio, the methanol & DME synthesis ability is higher, and the consumption ability of SAPO-34 reached saturation, the additional methanol & DME promoted H transfer reaction and caused further decrease in aromatics selectivity.

### Increasing the space-time yield of BTX

Zn_1.2_Cr(1)|6Mn4Zr/ZSM-5/SA(2 : 1 : 1)(8) exhibited a much higher CO conversion and BTX selectivity, further optimization were performed to enhance the productivity of BTX. Table 4 shows reaction results over Zn_1.2_Cr(1)|6Mn4Zr/ZSM-5/SA(*x* : 1 : 1)(8). The function of oxide components (6Mn4Zr in this research) has been investigated by other researches and proposed several mechanisms. Bao and Pan's group supposed the oxides as sites for generation of ketene which was regarded as primary intermediates over oxides alone. Wang *et al.* proposed a H backflow mechanism, which indicated the H species generated inside zeolites would diffuse back to oxides and released as H_2_ or participant in conversion of CO.^30^ However, in this research, neither the mechanism above was fully suitable. Without existence of 6Mn4Zr, the major products shifted to light paraffins, with C_5_^+^ paraffins, and CO conversion reached only 25.79%. Addition of 6Mn4Zr could provide another reaction pathway for activation of CO by participant in the C–C coupling over surface of 6Mn4Zr and provide higher oxygenates. Those oxygenates could promote the selectivity of total aromatics by avoiding H transfer reaction. However, with further increasing the proportion of 6Mn4Zr, it showed little impact on reaction results, which could be ascribed as the lack of zeolites. The too little amount of SAPO-34 and ZSM-5 limited consumption of methanol & DME thus resulted in standstill of CO conversion and aromatics selectivity.

**Table 4:** Reaction results over Zn_1.2_Cr(1)|6Mn4Zr/H-ZSM-5/SAPO-34 (*x* : 1 : 1)(8)

Mass ratio of 6Mn4Zr to zeolites^a^	CO conversion (%)	CO_2_ selectivity (%)	Organic products selectivity (%)						Space-time yield of BTX (mg g_cat_^−1^ h^−1^)
			CH_4_	Methanol & DME	C_2_–C_4_ paraffin	C_2_–C_4_ olefin	C_5_^+^	Aromatics (BTX)	
0 : 1	25.79	39.74	5.69	0.04	61.22	4.94	21.76	6.36 (3.09)	8.37
0.5 : 1	25.30	40.46	2.05	0.04	22.91	5.08	15.48	54.45 (20.08)	52.73
1 : 1	30.77	39.88	1.72	0.03	19.87	5.17	11.51	61.70 (29.30)	93.68
1.5 : 1	30.78	40.40	1.82	0.03	20.20	5.57	10.67	60.71 (29.48)	92.36
2 : 1	30.37	41.41	2.06	0.07	19.19	9.17	9.01	60.50 (28.80)	89.35

a Mass ratio of H-ZSM-5 : SAPO-34 was kept as 1 reaction condition: 400 °C, 3 MPa, H_2_/CO = 2, 3000 mL g_cat_^−1^ h^−1^.

Zn_1.2_Cr(1)|6Mn4Zr/ZSM-5/SA(2 : 1 : 1)(8) exhibited a much higher CO conversion and BTX selectivity, further optimization were performed to enhance the productivity of BTX. Table 4 shows reaction results over Zn_1.2_Cr(1)|6Mn4Zr/ZSM-5/SA(*x* : 1 : 1)(8). The function of oxide components (6Mn4Zr in this research) has been investigated by other researches and proposed several mechanisms. Bao and Pan's group supposed the oxides as sites for generation of ketene which was regarded as primary intermediates over oxides alone. Wang *et al.* proposed a H backflow mechanism, which indicated the H species generated inside zeolites would diffuse back to oxides and released as H_2_ or participant in conversion of CO.^30^ However, in this research, neither the mechanism above was fully suitable. Without existence of 6Mn4Zr, the major products shifted to light paraffins, with C_5_^+^ paraffins, and CO conversion reached only 25.79%. Addition of 6Mn4Zr could provide another reaction pathway for activation of CO by participant in the C–C coupling over surface of 6Mn4Zr and provide higher oxygenates. Those oxygenates could promote the selectivity of total aromatics by avoiding H transfer reaction. However, with further increasing the proportion of 6Mn4Zr, it showed little impact on reaction results, which could be ascribed as the lack of zeolites. The too little amount of SAPO-34 and ZSM-5 limited consumption of methanol & DME thus resulted in standstill of CO conversion and aromatics selectivity.

The influence of reaction conditions were further investigated and shown in Table 5–7. As shown in Table 5, CO conversion at 350 °C could reach 13.49% with aromatics selectivity of 71.77%. Compared with our previous work, this CO conversion is much higher. The increase in CO conversion could be benefited from addition of SAPO-34, as it maintained more HCPs, the conversion of intermediates was facilitated. When the reaction temperature was lower than 425 °C, CO conversion was remarkably increased with rising reaction temperature. However, further increasing reaction temperature from 425 °C to 450 °C, CO conversion only increased from 38.77% to 41.31%. The selectivity of total aromatics is more sensitive to reaction temperature, it decreased from 71.77% at 350 °C to 61.70% at 400 °C, then sharply decreased to 43.91% at 425 °C; meanwhile, the selectivity of light paraffins increased. At higher reaction temperature, the less increase of CO conversion should be limited by thermodynamic equilibrium of methanol & DME synthesis. CO was mainly activated over Zn_1.2_Cr and converted to methanol & DME, which is consumed by 6Mn4Zr/H-ZSM-5/SAPO-34; the consumption of methanol & DME was mainly catalyzed by HCPs in zeolites. However, the change of selectivity in products indicated that H transfer reaction were accelerated at high reaction temperature, which might result from the loss of HCPs. Without enough HCPs, the consumptions of methanol & DME were inhibited and thus caused thermodynamic limitation of methanol & DME synthesis over Zn_1.2_Cr.

**Table 5:** Reaction results over Zn_1.2_Cr(1)|6Mn4Zr/H-ZSM-5/SAPO-34 (2 : 1 : 1)(8) at different reaction temperature^a^

Reaction temperature (°C)	CO conversion (%)	CO_2_ selectivity (%)	Organic products selectivity (%)						Space-time yield of BTX (mg g_cat_^−1^ h^−1^)
			CH_4_	Methanol & DME	C_2_–C_4_ paraffin	C_2_–C_4_ olefin	C_5_^+^	Aromatics (BTX)	
350	13.49	39.22	1.04	0.04	11.96	4.42	10.77	71.77 (30.07)	43.00
375	21.76	40.75	1.13	0.03	16.15	4.24	12.25	66.20 (29.86)	67.15
400	30.77	39.88	1.72	0.03	19.87	5.17	11.51	61.70 (29.30)	93.68
425	38.77	40.59	2.47	0.02	34.72	4.96	13.86	43.97 (24.64)	99.02
450	41.31	39.80	4.16	0.01	48.12	6.04	13.44	28.22 (17.39)	75.45

a Reaction condition: 3 MPa, H_2_/CO = 2, 3000 mL g_cat_^−1^ h^−1^.

**Table 6:** Reaction results over Zn_1.2_Cr(1)|6Mn4Zr/H-ZSM-5/SAPO-34 (2 : 1 : 1)(8) under different reaction pressure^a^

Reaction pressure (MPa)	CO conversion (%)	CO_2_ selectivity (%)	Organic products selectivity (%)						Space-time yield of BTX (mg g_cat_^−1^ h^−1^)
			CH_4_	Methanol & DME	C_2_–C_4_ paraffin	C_2_–C_4_ olefin	C_5_^+^	Aromatics (BTX)	
1	15.02	34.76	3.30	0.05	21.15	22.02	14.57	38.92 (27.76)	47.50
2	25.67	39.85	3.36	0.05	22.59	7.58	13.66	52.76 (30.87)	83.19
3	30.77	39.88	1.72	0.03	19.87	5.17	11.51	61.70 (29.30)	93.68
4	39.95	39.95	3.00	0.07	26.94	3.17	12.05	54.77 (24.27)	101.58
5	44.15	39.80	3.38	0.09	29.06	2.43	12.79	52.25 (21.65)	100.36

a Reaction condition: 400 °C, H_2_/CO = 2, 3000 mL g_cat_^−1^ h^−1^.

**Table 7:** Reaction results over Zn_1.2_Cr(1)|6Mn4Zr/H-ZSM-5/SAPO-34 (2 : 1 : 1)(8) with different space velocity^a^

Space velocity (mL g_cat_^−1^ h^−1^)	CO conversion (%)	CO_2_ selectivity (%)	Organic products selectivity (%)						Space-time yield of BTX (mg g_cat_^−1^ h^−1^)
			CH_4_	Methanol & DME	C_2_–C_4_ paraffin	C_2_–C_4_ olefin	C_5_^+^	Aromatics (BTX)	
3000	30.77	39.88	1.72	0.03	19.87	5.17	11.51	61.70 (29.30)	93.68
2400	41.51	41.77	1.27	0.01	26.91	2.10	10.44	59.27 (31.37)	105.90
1800	45.94	41.45	1.47	0.01	28.61	1.96	10.87	57.08 (29.35)	82.69
1200	52.78	41.68	1.74	0.01	31.22	1.68	10.83	54.51 (26.97)	57.96
600	64.18	40.88	2.34	0.01	37.77	1.30	10.23	48.35 (22.70)	30.07

a Reaction condition: 400 °C, 3 MPa, H_2_/CO = 2.

As show in Table 6, CO conversion increased with increasing reaction pressure; however, at lower reaction pressure, the total aromatics selectivity achieved only 38.92% which was a much lower value with the comparation of 61.70% under 3 MPa. The BTX selectivity achieved maximum value of 30.87% at 2 MPa, while the total aromatics selectivity achieved maximum value of 61.70% at 3 MPa, moreover, the BTX space time yield reached maximum value of 101.58 mg g_cat_^−1^ h^−1^ at 4 MPa. The methanol & DME synthesis activity of Zn_1.2_Cr is much higher even at lower reaction pressure, thus CO conversion could reach 15.02% even at 1 MPa, however, the C–C coupling activity of 6Mn4Zr is relative lower which is caused by low partial pressure of CO. The C_2_–C_4_ olefins generated from SAPO-34 could not be consumed by 6Mn4Zr and H-ZSM-5, thus resulted in low total aromatics selectivity at 1 MPa. With increasing reaction pressure, the adsorption of CO over 6Mn4Zr were enhanced, promoting conversion of light olefins by C–C coupling. Oxygenates generated from C–C coupling over 6Mn4Zr could be further converted into aromatics, which is the reason for increase of total aromatics selectivity. Besides, increasing reaction pressure, the activity of methanol & DME synthesis is also promoted, and the generated abundant methanol & DME would cause methylation of BTX components; when further increasing reaction pressure, consumption ability of methanol & DME over SAPO-34 reached saturation, thus facilitated H transfer reaction and caused decrease in total aromatics selectivity.

**Fig. 4:**
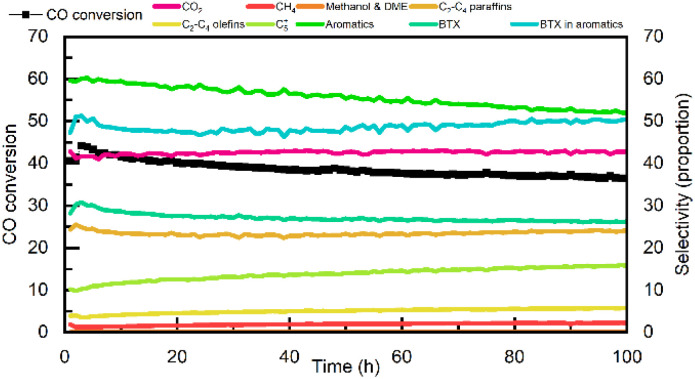
Stability evaluation of Zn_1.2_Cr(1)|6Mn4Zr/H-ZSM-5/SAPO-34 (2 : 1 : 1)(8).

Therefore, the optimal reaction condition was chosen as 400 °C, 3 MPa, 2400 mL g_cat_^−1^ h^−1^, H_2_/CO = 2. The stability of Zn_1.2_Cr(1)|6Mn4Zr/H-ZSM-5/SAPO-34 (2 : 1 : 1)(8) were evaluated at optimal condition, and the reaction results were shown in Fig. 4. The CO conversion rose slowly in the first 3 hours, while the total aromatic hydrocarbon selectivity gradually decreased, and the BTX component selectivity gradually increased. This indicated that HCPs began to be generated in the channels of SAPO-34. Both the STO study and the MTO study in the OX-ZEO indicated that there was an induction period at the beginning of the reaction, during which the accumulation of HCPs in the zeolites occurred mainly.^2,31–33^ After the induction period, the catalyst slowly lost its activity. During this process, the CO conversion rate, total aromatic selectivity, and BTX selectivity all decreased, but the proportion of BTX in the aromatics remained almost unchanged. Meanwhile, the selectivity of C_5_^+^ alkanes increased. The fact that the proportion of BTX in the total aromatics remained unchanged indicates that SAPO-34 still plays a major role in the consumption of intermediates (methanol & DME). Therefore, the catalyst deactivation is not related to SAPO-34. Similar to the previous research,^21,29^ the deactivation of Zn_1.2_Cr(1)|6Mn4Zr/H-ZSM-5/SAPO-34(2 : 1 : 1)(8) mainly occurred on the H-ZSM-5 component. Since methanol & DME are converted to C_2_–C_4_ olefins on SAPO-34, these olefins are further converted to oxygenates on 6Mn4Zr. Due to the fast generation of those oxygenates, coke deposition over external surface of H-ZSM-5 is accelerated and increased diffusion resistance, preventing the oxygenates adsorbed on 6Mn4Zr from desorbing and finally hydrogenated to C_5_^+^ paraffins.

## Conclusions

SAPO-34 were added into bifunctional catalyst composed of 6Mn4Zr and H-ZSM-5, and increased both CO conversion and aromatics selectivity. Addition of SAPO-34 increased the accumulated amount of HCPs in catalyst and thus promoted conversion of intermediates including methanol & DME. As methanol & DME were consumed mainly in SAPO-34, the methylation of BTX were thus inhibited and achieved higher BTX selectivity. Further, 6Mn4Zr/H-ZSM-4/SAPO-34 (denoted as 6Mn4Zr/H-ZSM-5/SAPO-34) were coupled with Zn_*X*_Cr, and achieved much higher CO conversion. The influence of Zn/Cr ratio, mass ratio of H-ZSM-5/SAPO-34, 6Mn4Zr/zeolite and reaction condition (including reaction temperature, pressure and space velocity) were investigated. 6Mn4Zr was found essential for generation of aromatics as light olefins could be converted into oxygenates over surface of 6Mn4Zr. Moreover, increasing the ability for methanol and DME generation could facilitate H transfer reaction and methylation reaction, resulting in decreased in selectivity of both aromatics and BTX components. The stability of Zn_1.2_Cr(1)|6Mn4Zr/H-ZSM-5/SAPO-34 (2 : 1 : 1)(8) was evaluated at 400 °C, 3 MPa, 2400 mL g_cat_^−1^ h^−1^, H_2_/CO = 2, after 100 h reaction, CO conversion could still maintain 36.49% with BTX space time yield of 76.36 mg g_cat_^−1^ h^−1^.

## Author contributions

Shiyu Liu: writing – original draft, data curation, conceptualization, methodology, investigation; Qiuyun Huang: data curation, methodology; Jie Wang: writing – original draft; Weihua Shen and Yunjin Fang: funding acquisition, supervision, writing – review and editing.

## Conflicts of interest

There are no conflicts to declare.

## Supplementary Material

RA-016-D5RA08666C-s001

## Data Availability

The authors confirm that the data supporting the findings of this study are available within the article and its supplementary information (SI). Supplementary information is available. See DOI: https://doi.org/10.1039/d5ra08666c.
