# Supplementary material for: Increase of light aromatics space time yield by addition of SAPO-34 into multifunctional catalyst
Source: RSC Adv. 2026 Jan 5;16(1):353–61. doi: 10.1039/d5ra08666c (PMC12766377; doi:10.1039/d5ra08666c)
Supplement: RA-016-D5RA08666C-s001 [file RA-016-D5RA08666C-s001.pdf]

Supplementary Information (SI) for RSC Advances.
This journal is © The Royal Society of Chemistry 2026

Increase of light aromatics space time yield by addition of SAPO-34 into multifunctional catalyst

Shiyu Liu[a], Qiuyun Huang[a], Jie Wang[a], Weihua Shen[a,*], Yunjin Fang[a,*]

[a]State Key Laboratory of Chemical Engineering and Low-Carbon Technology, School of Chemical Engineering, East China University of Science and Technology, Shanghai 200237, China.

*Corresponding Author

Email: whshen@ecust.edu.cn(Prof. Shen); yjfang@ecust.edu.cn(Prof. Fang)

Tel: +86-21-64252829

Table S1 XRF results of oxides and zeolites

Sample	Molar composition		
6Mn4Zr	Mn (mol%)	Zr (mol%)	Mn/Zr ratio
	59.35	40.65	1.46
	Zn (mol%)	Cr (mol%)	Zn/Cr ratio
Zn1.0Cr	27.19	46.19	0.94
Zn1.1Cr	29.19	42.90	1.08
Zn1.2Cr	31.94	42.38	1.20
Zn1.3Cr	32.86	39.69	1.32
H-ZSM-5(60)	Si (mol%)	Al (mol%)	
	98.32	1.68	
SAPO-34(0.1)	Si (mol%)	Al (mol%)	P (mol%)
	4.44%	43.54%	52.02%

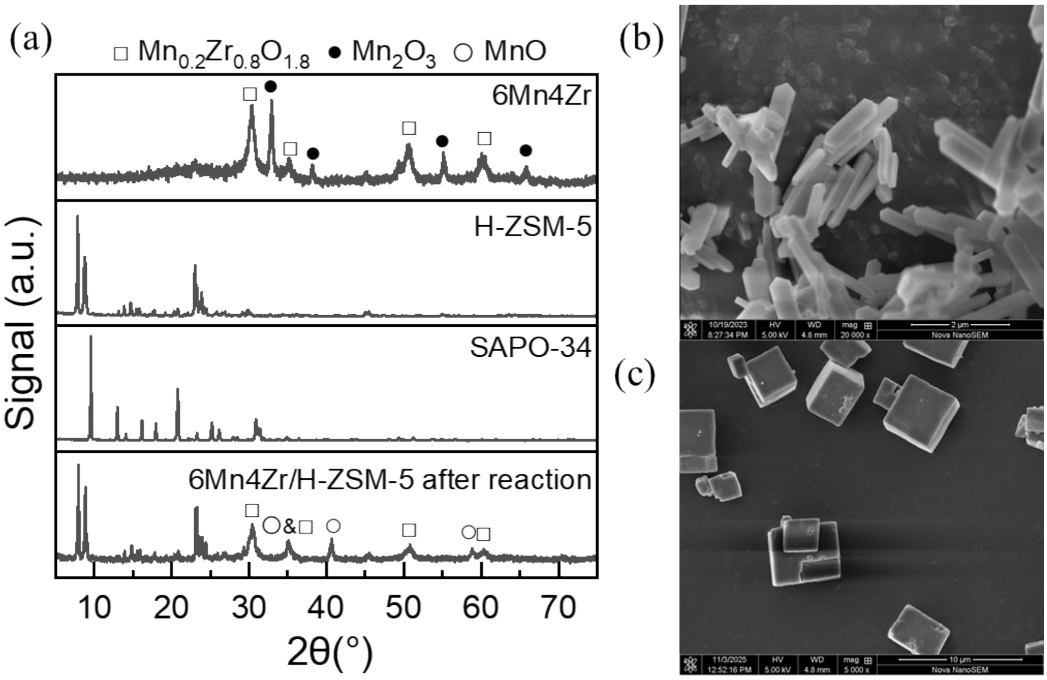

Figure S1. (a) XRD patterns of 6Mn4Zr, H-ZSM-5 and SAPO-34; (b) SEM figure of H-ZSM-5 and (c) SEM figure of SAPO-34.

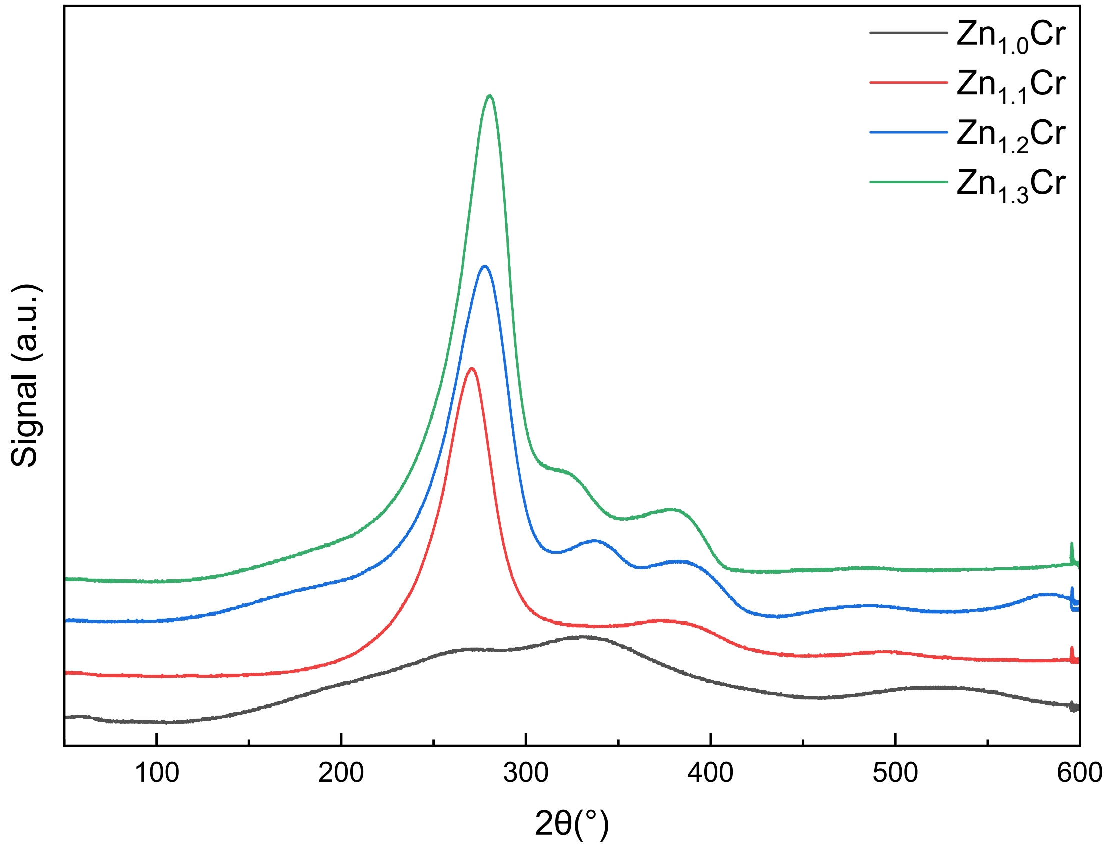

Figure S2. H_2-TPR profile of Zn_XCr.

Table S2. Quantification of XPS profiles of Zn_XCr

Oxides	Fresh oxides		Reduced oxides	
	Zn/Cr ratio (mol%)	Oxygen vacancy (mol%)	Zn/Cr ratio (mol%)	Oxygen vacancy (mol%)
Zn1.0Cr	0.32	15.95	0.34	23.07
Zn1.1Cr	0.45	21.51	0.47	26.97
Zn1.2Cr	0.56	22.26	0.59	28.99
Zn1.3Cr	0.63	25.35	0.68	30.39

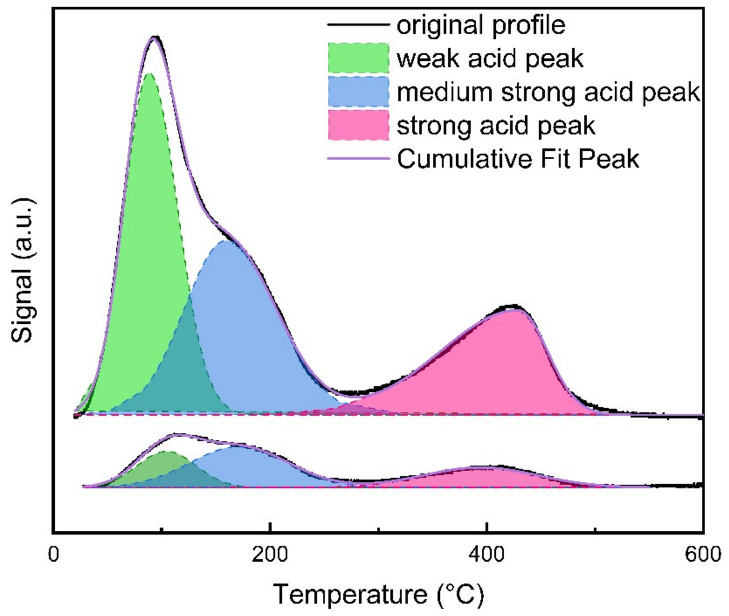

Figure S3. NH_3-TPD profile of zeolites.

Table S3 Quantification of acid sites in Figure S2

Zeolites	Acid sites distribution			Total acid sites
	Weak acid sites	Medium strong acid sites	Strong acid sites	μmol/g
H-ZSM-5	19.07%	52.09%	28.84%	73.45
SAPO-34	43.01%	31.56%	25.43%	720.41

Table S4 Quantification of HCPs in figure 3

Mass ratio of H-ZSM-5 to SAPO-34	Mass ratio at 230°C	Mass ratio at 800°C	Amount of HCPs
1:0	98.23	95.52	2.71
2:1	88.66	85.14	3.52
1:1	88.25	82.53	5.72
1:2	90.84	85.20	5.64
0:1	85.49	80.31	5.18

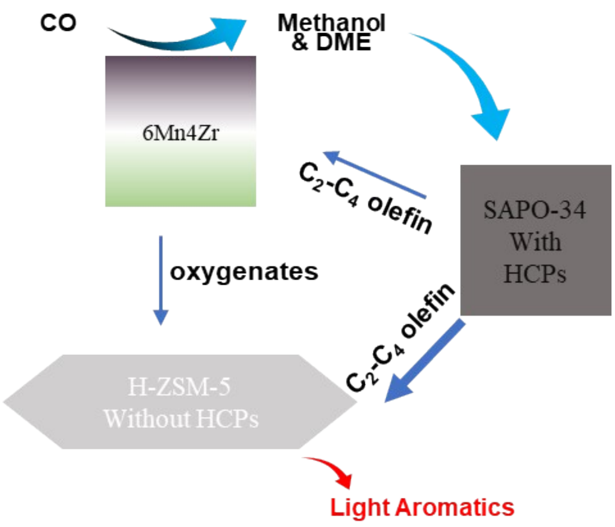

Scheme S1. Reaction mechanism over 6Mn4Zr/H-ZSM-5/SAPO-34

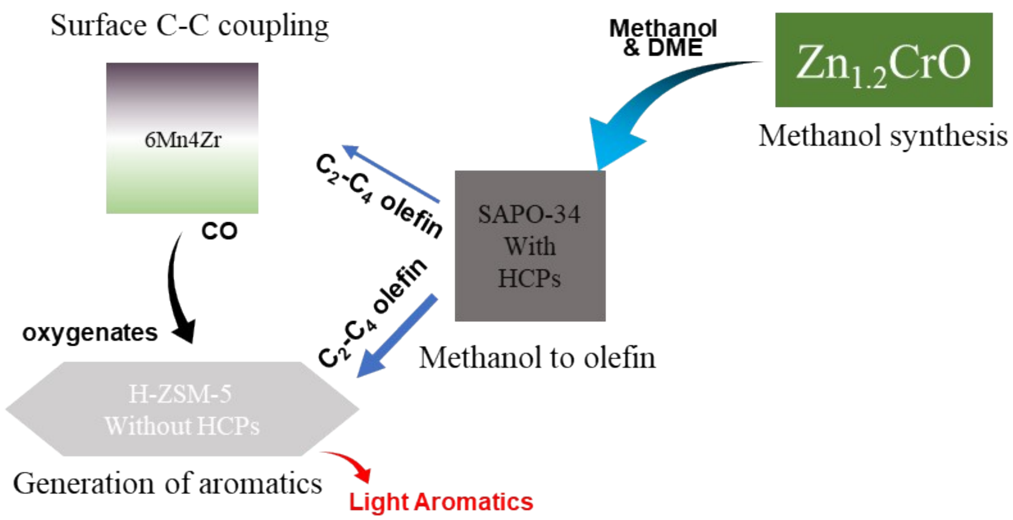

Scheme S2. Reaction mechanism over $Zn_{1.2}Cr$(*1*)|6Mn4Zr/H-ZSM-5/SAPO-34(2:1:1)(*8*)
